# Flow and detailed 3D morphodynamic data from laboratory experiments of fluvial dike breaching

**DOI:** 10.1038/s41597-019-0057-y

**Published:** 2019-05-13

**Authors:** Ismail Rifai, Kamal El kadi Abderrezzak, Sébastien Erpicum, Pierre Archambeau, Damien Violeau, Michel Pirotton, Benjamin Dewals

**Affiliations:** 1 Saint-Venant Laboratory for Hydraulics (LHSV), Chatou, France; 2 EDF R&D, National Laboratory for Hydraulics and Environment (LNHE), Chatou, France; 3 Hydraulics in Environmental and Civil Engineering (HECE), University of Liège, Liège, Belgium

**Keywords:** Natural hazards, Civil engineering, Databases

## Abstract

This paper presents a dataset obtained from fifty four laboratory experiments of the breaching of fluvial dikes due to flow overtopping. Data were collected on two complementary experimental setups, each consisting of a main channel representing the river, an erodible lateral dike and a floodplain. The dataset covers seven test series, involving varying hydraulic boundary conditions (*e*.*g*. inflow discharge, downstream boundary conditions), main channel dimensions, as well as bottom and dike material. The following experimental data were produced: time series of water levels in the main channel, time series of flow discharges in the main channel and through the breach, as well as high resolution 3D reconstructions of the breach geometry during its expansion. The latter measurements were performed using a novel non-intrusive laser profilometry technique developed for this research. Reuse of the collected data will support efforts to improve our understanding of the physical processes underpinning fluvial dike breaching. It will also enable benchmarking the accuracy of conceptual or detailed numerical models for the prediction of dike breaching, which is central to flood risk management.

## Abstract (summary)

**Table:** 

Design Type(s)	modeling and simulation objective • factorial design
Measurement Type(s)	levee
Technology Type(s)	Modeling_and_Simulation
Factor Type(s)	Regulator Device • fluid flow rate • Dimension
Sample Characteristic(s)	laboratory environment

Machine-accessible metadata file describing the reported data (ISA-Tab format)

## Background & Summary

Fluvial dikes, *i*.*e* embankment levees, along river banks, are built to channelize the flow and limit lateral riverbed migration, or as defence structures against floods. Dike failure during a flood event may lead to casualties and major damage in the protected areas, which can be more severe than that of equivalent floods without the presence of dikes. Understanding the dike breach processes and the potential consequences of dike failure induced floods is a major concern, as sustained demographic and economic development is witnessed in dike-protected areas^1^ and these areas can be the location of highly sensitive and strategic assets. However, despite the major relevance of the topic for public safety, and despite several leading research groups and engineering entities seeking the development of reliable dike breaching models, major uncertainties regarding the physical processes are still hampering the proper consideration of fluvial dike breaching for flood risk analysis and management^2,3^.

Frequent exposure to flow actions (*e*.*g*. high or rapidly varying water levels, piping, and riverside erosion), lack of maintenance, inadequate rehabilitation works and animal burrows^4^ contribute to dike weakening and increase the risk of structural failures. Different dike failure mechanisms can be identified, which can be categorized as^5,6^: (i) Instability, referring to defects on dike structural integrity when soil particle movement active strengths exceed resistant strengths^7^, (ii) Internal erosion through the dike and/or foundation body due to seepage flows, (iii) External erosion, which broadly encompasses mechanisms involving wearing of the structure surface due to floods, waves, wind, or any other natural process^8^. Flow overtopping, generating external erosion, is identified as the most frequent cause of dike failure^9–11^. Dike overtopping occurs if the river discharge exceeds the design value of the dike during a flood event, or, broadly, if the water level exceeds the dike crest or the flow overtops a weak dike segment.

The flow over the dike crest can trigger external erosion, eventually leading to breaching of the dike and catastrophic damage on the adjacent floodplain. Therefore, the breach growth dynamics and subsequent breach flow hydrograph have to be accurately estimated as they are key inputs for flood risk analysis and management. The present dataset is the outcome of a research project aiming at improving our understanding of the physical processes underpinning breach expansion and breach hydraulics in the case of fluvial dike failures^3,12^. A comprehensive test program was conducted (54 tests) on two experimental models, shedding light on dike breaching dynamics in various realistic configurations, including varying: (i) channel flow conditions, (ii) channel dimensions, (iii) floodplain confinement level, (iv) bottom erodibility, and (v) dike material composition. Each test was densely monitored using a 3D continuous reconstruction (*i*.*e*. digital elevation model) of the breach geometry, which allows a full depiction of the breach expansion stages. Water levels in the main channel were measured, allowing the estimation of the flow discharge through the breach.

The present dataset^13^ complements and comprises findings, counting datasets, discussed in two previous research papers: (i) the experiments conducted by Rifai *et al*.^3^ which focuses on the channel flow condition effects on dike breaching and (ii) the work conducted by Rifai *et al*.^12^ investigating tailwater effects, *i*.*e*. water level increase on the floodplain side, on breach expansion dynamics. Details of the experimental method and scientific analyses of the present dataset were also presented in the PhD thesis of Rifai^14^.

On one hand, the generated dataset enables a thorough understanding of the mechanisms involved in breach expansion, and, on the other hand, it constitutes a sound database for testing modelling hypotheses and validating numerical models.

## Methods

### Experimental models

**Fig. 1:**
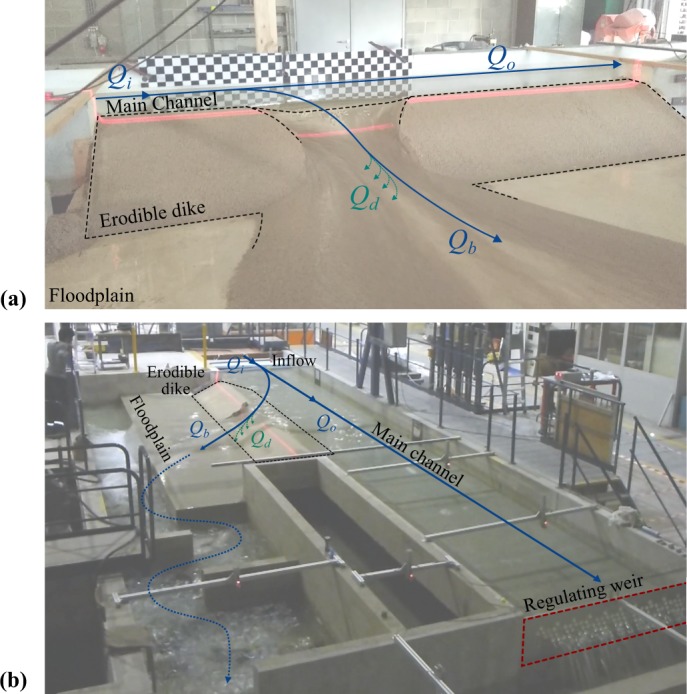
Experimental models. (**a**) ULiège and (**b**) LNHE. *Q*_*i*_ is the inflow discharge, *Q*_*b*_ the breach discharge, *Q*_*d*_ the drainage discharge, and *Q*_*o*_ the main channel outflow discharge.

**Fig. 2:**
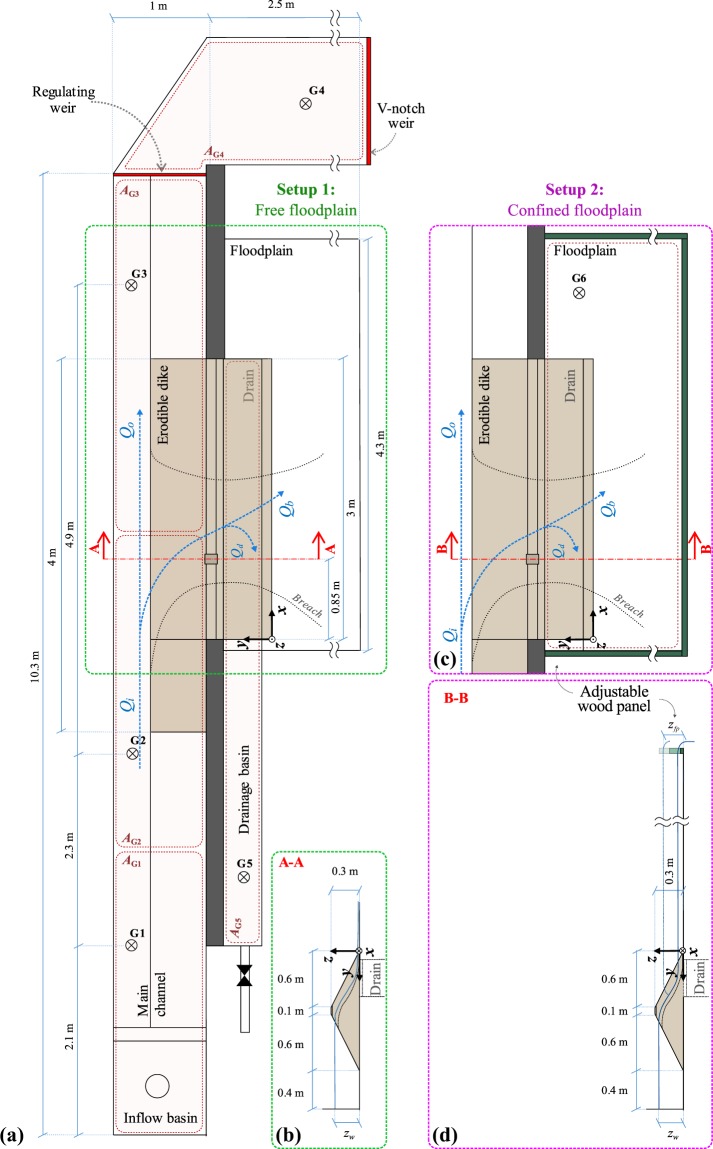
ULiège experimental facility. (**a**) Plan view with a free floodplain, (**b**) side view (cut A-A), (**c**) plan view with a confined floodplain, and (**d**) corresponding side view (cut B-B).

**Fig. 3:**
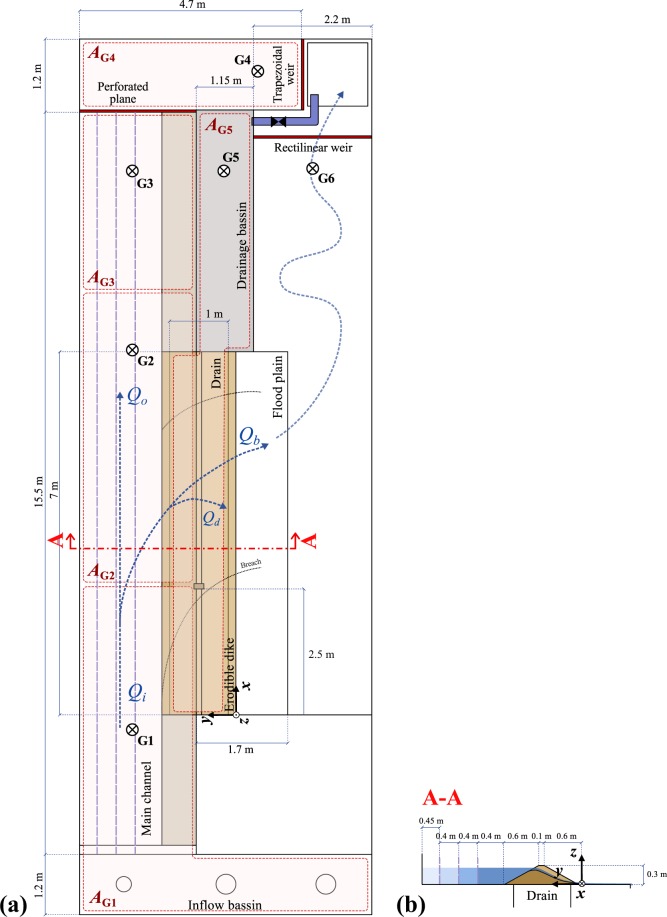
LNHE experimental facility. (**a**) Plan view, and (**b**) side view with different main channel widths (cut A-A).

Two laboratory facilities were designed and built (Fig. 1). Experiments consisted in provoking the overtopping of the main channel flow over the dike crest at a designated spot (*i*.*e*. initial notch) and observing subsequent dike erosion and breach expansion. The first experimental model (referred herein as ULiège model) was built at the Engineering Hydraulics Laboratory of University of Liège (Figs 1a and 2). The second one (*i*.*e*. LNHE model) was built at the National Laboratory for Hydraulics and Environment of EDF-R&D (Figs 1b and 3). These two facilities differ in the main channel dimensions (width, length) and the dike length. The experimental models were intended to be complementary in their design, which allowed conducting various test series while investigating different parameters. Each model consisted of a main straight channel with a long side opening toward a floodplain. This side opening was closed with sand, representing a trapezoidal shaped fluvial dike separating the main channel and the protected floodplain. In all tests, the dike was *h*_*d*_ = 0.3 m high, the crest was *l*_*dc*_ = 0.1 m wide and the inner and outer dike face slopes were *S*_*i*_ = *S*_*o*_ = 1:2 (V:H); the bottom dike width was *l*_*d*_ = *h*_*d*_/*S*_*i*_ + *h*_*d*_/*S*_*o*_ + *l*_*dc*_ = 1.3 m. The bottom of the main channel and the floodplain were at the same level and can be made of erodible material.

The inflow discharges *Q*_*i*_, into the main channel was kept steady throughout each test. In the ULiège model, three different regulating systems were tested at the main channel outlet: (i) perforated plane, (ii) rectilinear weir, and (iii) sluice gate. Each regulating system was calibrated to insure that the main channel water surface, prior to breaching, was at the crest level for the considered inflow discharge *Q*_*i*_. Therefore, for each test the number of holes in the perforated plane, the weir crest level, or the sluice gate level were adjusted. In the LNHE model, only a perforated plane was used.

To ensure dike stability prior to overtopping, the seepage flow was limited by installing a drainage system at the dike bottom (see sections A-A and B-B in Fig. 2, section A-A in Fig. 3). The drainage system consisted of a 4 cm-thick layer of dike material wrapped in a geotextile that was placed on a coarse grid. To trigger overtopping, a 0.02 m deep, 0.1 m wide initial notch was cut in the dike crest at 0.85 m and 2.55 m from the dike upstream end in the ULiège model and LNHE model, respectively (Figs 2 and 3).

In the ULiège model, the main channel was *L*_*mc*_ = 10 m long, *l*_*mc*_ = 1 m wide; the erodible dike was *L*_*d*_ = 3 m long and the floodplain was 4.3 m × 2.5 m. In most tests the flow through the breach was discharged freely from the floodplain without any storage change nor tailwater effects (Fig. 2a and Online-only Table 1, test series A, B, D and E). In some tests the floodplain was confined with wood panels to prescribe a predefined floodplain water level *z*_*fp*_ (Fig. 2c and Online-only Table 1, test series C). Two filling non-cohesive, uniform materials were tested for the dike construction, namely uniform coarse sand with median diameter *d*_50_ = 1 m or 1.67 mm (Table 1, Materials 1 and 2). For selected tests the water surface in the main channel was controlled by different downstream regulating systems (perforated plane, rectilinear weir, sluice gate).

**Table 1:** Properties of the material used for dike breaching experiments.

	Material F (fine sand)	Material 1 (coarse sand)	Material F1	Material F2	Material F3	Material 2 (coarse sand)
Median diameter *d*_50_ (mm)	0.24	1.03	0.98	0.96	0.91	1.67
Volumetric ratio of fine sand (%)	100	0	10	20	30	0
Density *ρ*_*s*_ (kg/m^3^)	2485	2470	2478	2503	2510	2470
Bulk density *ρ*_*b*_ (kg/m^3^)	1540	1556	1710	1877	1883	1507
Porosity *p* (−)	0.38	0.37	0.31	0.25	0.25	0.39
Friction angle dry *φ* (°)	28-30	28	—	30	—	—
Friction angle wet *φ*_*w*_ (°)	—	55	—	—	—	—
Tilt angle (°)	32	30	—	29	—	—

The LNHE model channel was *L*_*mc*_ = 17.9 m long and allowed adjusting the channel width *l*_*mc*_; three values were tested, namely 1.4 m, 1.8 m and 2.25 m. The channel side opening, *i*.*e*. dike length, was *L*_*d*_ = 7 m long and the free floodplain 7 m × 1.7 m. Different dike materials were tested, namely Material 1 (*i*.*e*. uniform coarse sand) and three other heterogeneous compositions consisting of Material 1 mixed with fine sand (Material F) of *d*_50_ = 0.24 mm. These three mixtures were made according to the volumetric fine sand ratios of 10%, 20%, and 30%, respectively (Table 1, Materials F1–F3). Finally, the LNHE model allowed conducting tests with erodible channel and floodplain bottoms, composed of the same material as the dike (Online-only Table 1, test series G); the moveable bed layer was 0.10 m thick.

Online-only Table 1 gives an overview of the overall test program, whereas Table 1 summarizes properties of the tested dike materials. In total, 54 tests were performed, 30 tests on the ULiège model and 24 on the LNHE model. Tests can be categorized in 7 series:

- Series A focuses on the effect of the inflow discharge *Q*_*i*_,
- Series B focuses on the effect of the main channel downstream boundary condition,
- Series C investigates the effect of the floodplain confinement,
- Series D investigates the effect of the grain size of the dike material,
- Series E focuses on the effect of the main channel size,
- Series F investigates the effect of fine sediments and associated apparent cohesion,
- Series G highlights the effect of bed changes in the channel and floodplain.

### Test procedure

Dike construction consisted in stacking and compacting the material in the channel side opening. Compaction was performed manually to avoid any structural defects. A template of the trapezoidal cross-section was then swiped along the longitudinal axis (*i*.*e*. *x*-axis) to shape the dike and remove excess material before setting the initial notch over which flow overtopping was initiated. At the test start, the main channel was filled progressively with a discharge *Q*_*i*0_ equal to about 75% of the nominal test inflow discharge *Q*_*i*_. For tests with a rectilinear weir, *Q*_*i*0_ ≈ 0.5 × *Q*_*i*_ to avoid overtopping of the initial notch during the “filling phase”. The inflow was provided by pumping from a tank, which formed a closed circuit with the experimental model. The inflow discharge *Q*_*i*_ was adjusted *via* a frequency converter and using valves. Once the flow stabilized, the dike and drainage system were inspected. The inflow discharge was then increased to the test nominal value *Q*_*i*_. The main channel water level increased up to overtop the dike crest at the initial notch triggering subsequent external erosion and dike breaching.

The material was reused after each test except of test series F which were conducted using fine sand and coarse sand mixtures. To check the validity of such reuse, the material grain size distribution was measured from three samples: one from the original material (supplier record), one from the dike before conducting the test, and one after conducting 20 tests. Reuse of the homogeneous material was deemed consistent as no significant grading was observed.

### Water level measurements

Water levels were recorded continuously at fixed locations (G1 to G6, Figs 2 and 3) using ultrasound sensors. Two sensors, mic + 35/IU/TC and mic + 130/IU/TC by Microsonic, were used depending on the range of water elevation variability.

### Flow discharge calculation

The inflow discharge *Q*_*i*_ was measured using an electromagnetic flowmeter. The main channel outflow discharge *Q*_*o*_ was estimated from the discharge passing through a measuring weir *Q*_*o*,*mw*_, using a V-notch weir in the ULiège model and a trapezoidal weir in the LNHE model. *Q*_*o*_ was evaluated from the following mass balance in the control volume between the main channel downstream regulating weir and the measuring weir:

1 $${Q}_{o}\left(t\right)={A}_{G4}\times d{z}_{w,o}/dt+{Q}_{o,mw}\left(t\right)$$

with *t* the time, *A*_G4_ the water surface area of the control volume, and *z*_*w*,*o*_ the water level measured at gauge G4.

The breach discharge *Q*_*b*_ was determined using the same approach (*i*.*e*. prismatic routing)^15–19^, adapted to the control volume of the main channel:

2 $${Q}_{b}\left(t\right)={Q}_{i}\left(t\right)-{Q}_{o}\left(t\right)\,-\,{Q}_{d}\left(t\right)-({A}_{G1}+{A}_{G2}+{A}_{G3})\times d{z}_{w}/dt$$

with *z*_*w*_ = (*A*_G1_*z*_G1_ + *A*_G2_*z*_G2_ + *A*_G3_*z*_G3_)/(*A*_G1_ + *A*_G2_ + *A*_G3_) a weighted average of water levels *z*_G1_, *z*_G2_, and *z*_G3_ at G1, G2, and G3, respectively, with *A*_G1_, *A*_G2_, and *A*_G3_ as main channel surface areas associated with G1, G2, and G3, respectively (Figs 2 and 3). On the ULiège model, the drainage discharge *Q*_*d*_ was computed by a prismatic routing on the drainage reservoir placed under the dike, *i*.*e*. *Q*_*d*_ = *A*_G5_ × d*z*_*w*,*dr*_/d*t*, with *z*_*w*,*dr*_ the water level in the reservoir tank bellow the dike, and *A*_G5_ the reservoir surface area. When the drainage reservoir was full, a drain valve was opened. During the tank emptying phase, measurements were not collected. The drainage discharge was expected to have small variations once the breaching is established. Thus, linear interpolation between measurements was deemed satisfactory to estimate *Q*_*d*_. For the LNHE model, continuous measurements of *Q*_*d*_ were not feasible due to the limited storage capacity of the drainage tank. *Q*_*d*_ was deduced by prismatic routing at the test start only, and was considered constant for the test duration.

### Dike 3D reconstruction

Dike breaching was monitored by a laser profilometry technique (LPT), a non-intrusive method consisting in sweeping a laser plan (emitted by a laser line projector) over the dike and processing the images recorded by a single video camera located above the dike. The reconstruction relies on the Direct Linear Transformation (DLT) algorithm for camera calibration, accounting for optical and decentring distortion^19^. Three-dimensional (3D) object coordinates of the laser profiles on recorded frames were deduced using the DLT and the laser plan position in the object coordinates. Subaqueous laser profiles (*i*.*e*. laser profiles incident on submerged areas) were affected by a bias due to light refraction at the free surface. This bias was corrected assuming an abstract prismatic representation of the water free surface and using Snell-Descartes law^20^. Combining all generated profiles of one laser sweeping, a 3D referenced point cloud of the dike was constructed. One laser sweeping lasted between 1.5 to 5 seconds. The laser sweeping frequency was set depending on the breaching stage, with rapid sweepings at fast breach expansion stages and prolonged sweeping for slower stages. Each point cloud resulting from one sweeping was considered as an instantaneous representation of the dike geometry. Filtering was applied to the final point clouds to remove outliers and to reduce noise. The final point clouds were then projected onto a 1 cm × 1 cm grid. Additional details on the LPT are presented by Rifai *et al*.^21^ and Rifai^14^.

**Fig. 4:**
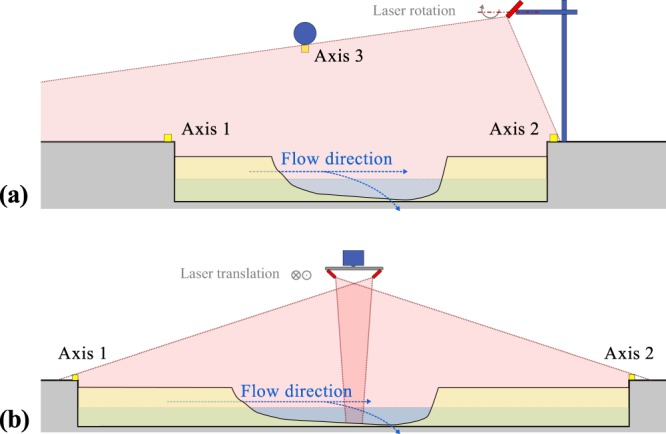
Vertical views of the laser sweeping setup. (**a**) ULiège model, and (**b**) LNHE model.

The laser profilometry setups were similar on both the experimental models. The video recording was performed with similar cameras set on a Full HD resolution (1920 × 1080 pixels) at 60 frames per second. The laser sweeping on the ULiège model was performed by a rotating laser projector (Fig. 4a). On the LNHE model, two laser projectors were used to cover the full 7 m dike length. The laser projectors were fixed to an automated sliding rail system (Fig. 4b).

## Data Records

### Data structure

**Fig. 5:**
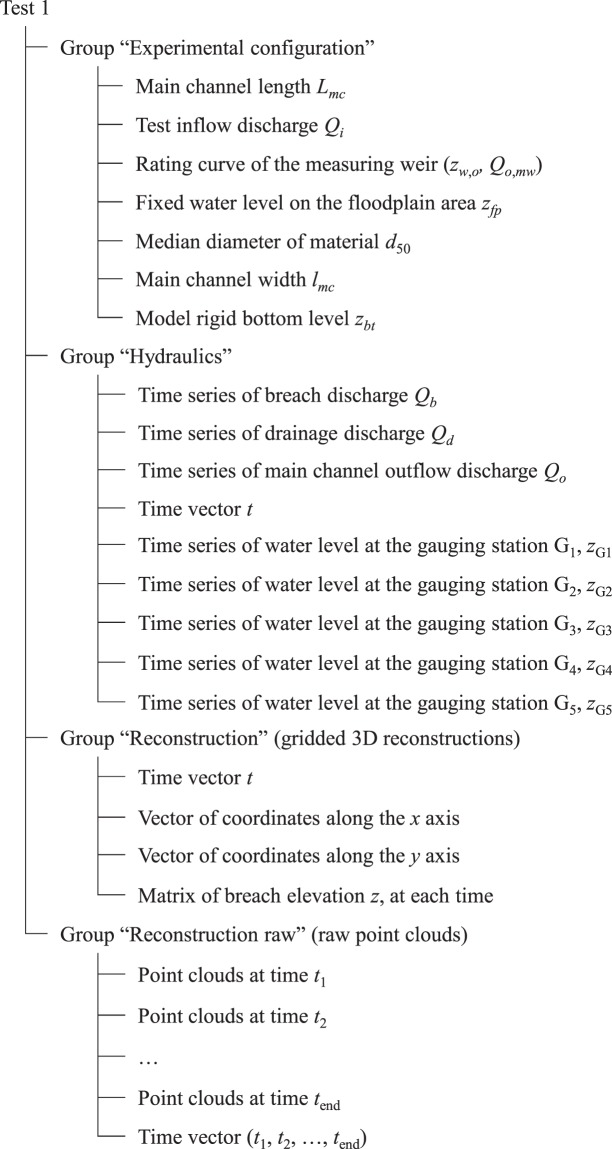
Example of data structure, corresponding to Test 1.
- “<*test name*>.h5” file containing the test full data. The data file is under the HDF5 format (Hierarchical Data Format 5). HDF5 libraries exist for different programing languages (*e*.*g*. Python, Matlab, Fortran, C, and R). More details can be found on the HDF5 website (https://support.hdfgroup.org/HDF5/),
- “<*test name*>_Exp_config.txt” file which summarises the experimental configuration and test boundary conditions. This file allows an easy reading of the experimental configuration data stored in the HDF5 file,
- “<*test name*>_hyd.png”, “<*test name*>_PC_raw.gif”, and “<*test name*>_DEM.gif” present images and animated illustrations of the test results: time series of hydraulic variables (water levels and discharges), point clouds reconstructions, and rasterized reconstructions, respectively.

The present dataset^13^ is structured as presented in Online-only Table 1. Data corresponding to each test is stored in a directory named according to the test number. The file “tree.txt” presents a depth-indented listing of test directories and data stored within. Each test directory comprises:

- “<*test name*>_data_structure.txt” file which presents the tree structure and content of the test data full file “<*test name*>.h5” (see ‘Supplementary File 1’ for an example of such file, and Fig. 5 for a sketch of the corresponding data structure for Test 1),

Data stored in the HDF5 files is structured flowing a directory, subdirectory, and file tree structure, corresponding to groups, subgroups and datasets, respectively (https://support.hdfgroup.org/HDF5/). The data is organized under four groups, namely: (i) experimental configuration, which summarizes the model dimensions and test boundary conditions, (ii) hydraulic variables, including, the measured water levels at different gauging locations as well as discharges, (iii) raw 3D point cloud reconstructions, and (iv) rasterized reconstructions.

In addition, a Python script, “read_datah5.py”, is provided to serve as an example of data retrieving and display from the HDF5 files. The script can be used to start processing and analysis of data. The “Extract_example.mp4” video is a screen recording showing how the retrieving and display of the data, using the provided Python script, works. The videos “Test_6.mp4” and ‘Test_49.mp4” are stop-motion videos recording during the experiments for tests conducted on the ULiège model and LNHE model, respectively.

### Notations and units

Datasets in the HDF5 are labelled following the physical variable name. Table 2 lists names of variables and associated units.

**Table 2:** Dataset notations and units.

	Label in the dataset	Variables		Units
Experimental configuration	Q_i	*Q*_*i*_	Test inflow discharge	(m^3^/s)
	L_mc	*L*_*mc*_	Main channel length	(m)
	l_mc	*l*_*mc*_	Main channel width	(m)
	Z_fp	*z*_*fp*_	Fixed water level on the floodplain area	(m)
	z_bt	*z*_*bt*_	Model rigid bottom level	(m)
	d_50	*d*_50_	Median diameter of material	(m)
Hydraulic variables	t	*t*	Time	(s)
	z_G*i*	*z*_G *i*_	Water level at the gauging station G_*i*_	(m)
	Q_b	*Q*_*b*_	Breach discharge	(m^3^/s)
	Q_d	*Q*_*d*_	Drainage discharge	(m^3^/s)
	Q_o	*Q*_*o*_	Main channel outflow discharge	(m^3^/s)
Reconstructions	t	*t*	Time	(s)
	x	*x*	Coordinate on the *x* axis	(m)
	y	*y*	Coordinate on the *y* axis	(m)
	z	z	Coordinate on the *z* axis	(m)

## Technical Validation

### Hydraulic variables

Water levels were measured using ultrasound probes. The probe measurement accuracy was ±1%. Record frequency was set to 100 measurements per second for the ULiège model and 10 measurements per second for the LNHE model. Measurements were then processed and outliers were deleted based on a moving average and standard deviation thresholding. For the sake of visibility and further processing, the measured signals were smoothed using a low-pass filter, namely the Savitzky & Golay^22^ filtering function of Matlab 2014b (sgolayfilt). The polynomial order and the frame length parameters of the functions were adjusted depending on the measurement frequency. The use of the Savitzky & Golay^22^ filter better preserves the distribution features (*i*.*e*. position and width of the extrema, area below the curve) in comparison to averaging techniques^22^.

**Fig. 6:**
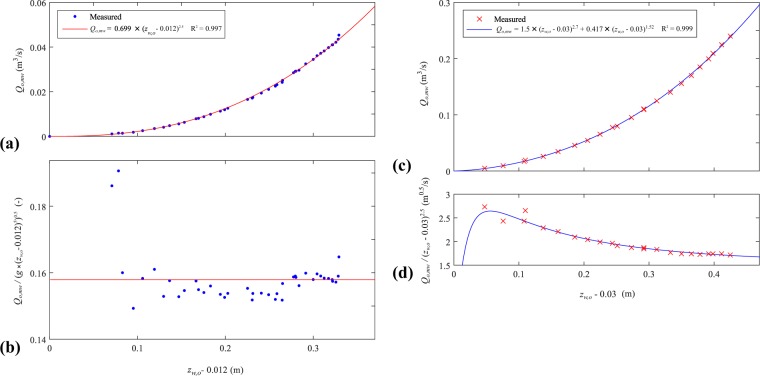
Rating curves at the measuring weirs. (**a**,**b**) V-notch weir used in the ULiège model, (**c**) and (**d**) Trapezoidal weir used in the LNHE model.

Water level measurements were used to compute the channel outflow, breach, and drainage discharges (*cf*. flow discharge calculation). The channel outflow discharge *Q*_*o*_ at the regulating weir was deduced from the water level measured at G4 and the rating curve of the measuring weir (Eq. 1). The rating curve of each measuring weir was calibrated and corresponding adjustment formula were proposed (Fig. 6). A comparison between the computed outflow discharges *Q*_*o*_ and those deduced from the rating curve of a calibrated perforated plane showed a maximum deviation below 10% of the test inflow discharge *Q*_*i*_, whereas the root mean square error represented 3.5% of *Q*_*i*_.

Mass balance on the main channel enabled the estimation of the breach discharge *Q*_*b*_ (Eq. 2), with the inflow discharge *Q*_*i*_ measured at the inflow pipe with an electromagnetic flow meter of ±4% accuracy. The water surface rippling induced physical disturbances on the water level measurements. The breach discharge *Q*_*b*_ computed from Eq. 2 was therefore smoothed using the Savitzky & Golay^22^ filtering function.

### Dike geometry reconstructions

**Fig. 7:**
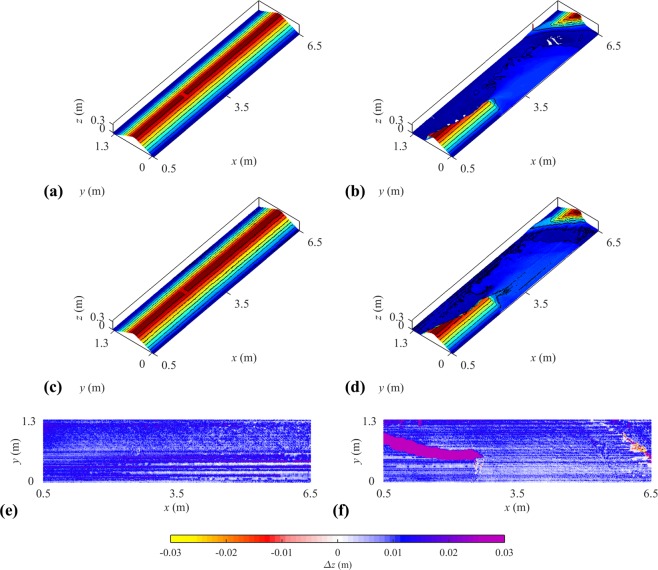
LNHE Model - Comparison of 3D reconstructions. (**a**) FARO Focus-3D of the initial dike, (**b**) FARO Focus-3D of the final dike, (**c**) LPT of the initial dike, (**d**) LPT of the final dike, (**e**) is the difference between (**a**,**c**,**f**) is the difference between (**b**,**d**).

Two additional 3D reconstructions were conducted with a laser scanner (FARO Focus-3D) to assess the overall validity of the LPT reconstructions. Scans were done on the initial and final dike geometries in the LNHE model (Fig. 7a–d). Each scan required up to 30 minutes and placing the laser scanner at two different locations and merge the generated point clouds to reduce blind spots. Figure 7e,f show differences between the laser scanner and LPT methods. Blind spots uncovered by the LPT were the main source of discrepancies between the reconstructions.

Other tests were conducted on the ULiège model with a plastered partially breached dike to assess the validity of the refraction correction module on a dike breaching experiment and the validity of the synthetic prismatic water surface assumption. Noticeable improvement of the results were obtained compared to the non-corrected reconstructions, although the refraction correction was slightly overestimated in some areas.

**Fig. 8:**
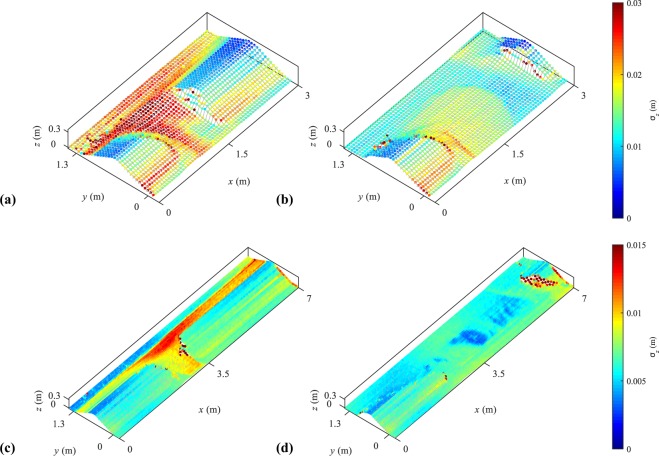
Uncertainties in the LPT reconstruction in the ULiège model. (**a**) Partially submerged dike and (**b**) dry dike, and in the LNHE model for (**c**) partially submerged dike and (**d**) dry dike.

Other sources of uncertainties in the LPT reconstruction scheme include^21^: (i) calibration of the camera based on the location of image calibration points as well as (ii) physical calibration points, (iii) extraction of the laser profiles by image filtering and processing, (iv) localization on the images of the intersection of the projected laser sheet with the laser identification axis, and (v) measurements and approximation of the main channel water surface levels used in the refraction correction steps. These uncertainty sources were quantified and propagated on four typical reconstructions: intermediate breach stages, involving refraction correction, and final reconstruction of a dry model. Results in Fig. 8 show the standard deviation of reconstruction uncertainties. It can be noted that the highest uncertainties come from the refraction correction as, in the partially submerged cases (Fig. 8a,c), the highest uncertainties are located in the submerged areas (*y* > 0.7 m and in the breach). In the non-submerged areas, uncertainties top at *σ*_*z*_ ≈ 0.02 m and ≈0.01 m on the ULiège model and LNHE model, respectively.

### Data usage caution

Note that, out of the 54 tests conducted, five of them (Tests 6, 37, 41, 42, and 54) have incomplete data. In Test 6, correction for refraction could not be performed. In Test 37, the camera did not operate between *t* = 1590 s and 3570 s. During Test 41, the ultrasound sensors did not operate between *t* ≈ 1000 s and 4600 s, liner interpolation of the water level time series was deemed sufficient in this case. Interpolated water level time series were then used for discharge calculation and water surfaces used for refraction bias correction. In Test 42, data from the ultrasound sensor G2 could not be processed. Finally, in Test 54, with the highest inflow discharge combined with an erodible bottom, the dike geometry reconstruction was affected by strong free surface variations due to flow turbulence.

## Usage Notes

This dataset is intended to bring insight into fluvial dike breaching processes and dynamic under various conditions. This dataset can be used to improve standardly used conceptual or numerical models to enable a comprehensive grasp of flow and erosion characteristics occurring throughout the different stages of the breaching process in a fluvial configuration (*e*.*g*. effects of saturation and apparent cohesion, secondary currents adjusted to abrupt flow deviation).

Another use of the dataset can include derivation of a simplified proxy representation of the breach geometry and expansion processes. This is relevant to practitioners as it can represent a more efficient implementation of dike breaching process in large scale numerical models used for flood risk assessment studies.

## Supplementary Information

### ISA-Tab metadata file

Download metadata file

### Supplementary information

Supplementary File 1

## Data Availability

The laser profilometry technique algorithm was developed on Matlab 2014b. Further details on the geometry 3D reconstruction and the algorithm structure are given by Rifai *et al*.^21^ and Rifai^14^.

## Online-only Table

**Online-only Table 1:** Overall test program

	Test series	Test n°	*Q*_*i*_ (m^3^/s)	*l*_*mc*_ (m)	*L*_*mc*_ (m)	Inlet Froude number F_*i*0_ (−)	Downstream boudary	*z*_*fp*_ (m)	*z*_*bt*_ (m)	Material
**ULiège model**	**A**	1	0.020	1	8	0.066	Perf.	0	0	Material 1 (uniform coarse sand *d*_50_ = 1.03 mm)
		2	0.021	1		0.070				
		3	0.028	1		0.093				
		4	0.030	1		0.100				
		5	0.031	1		0.103				
		6	0.040	1		0.133				
		7	0.040	1		0.133				
		8	0.040	1		0.133				
		9	0.041	1		0.136				
		10	0.047	1		0.156				
		11	0.050	1		0.166				
		12	0.051	1		0.169				
		13	0.055	1		0.182				
		14	0.057	1		0.189				
	**B**	15	0.030	1	8	0.100	Rec.	0	0	Material 1 (uniform coarse sand *d*_50_ = 1.03 mm)
		16	0.042	1		0.140				
		17	0.040	1		0.133				
		18	0.031	1		0.103	Slu.			
		19	0.041	1		0.136				
		20	0.040	1		0.133				
	**C**	21	0.041	1	8	0.136	Perf.	0.05	0	Material 1 (uniform coarse sand *d*_50_ = 1.03 mm)
		22	0.040	1		0.133		0.10		
		23	0.041	1		0.136		0.15		
		24	0.041	1		0.136				
		25	0.041	1		0.136		0.20		
		26	0.041	1		0.136				
	**D**	27	0.020	1	8	0.066	Perf.	0	0	Material 2 (uniform coarse sand *d*_50_ = 1.67 mm)
		28	0.031	1		0.103				
		29	0.040	1		0.133				
		30	0.050	1		0.166				
**LNHE model**	**E**	31	0.038	1	18	0.126	Perf.	0	0	Material 1 (uniform coarse sand *d*_50_ = 1.03 mm)
		32	0.046	1		0.153				
		33	0.054	1.4		0.108				
		34	0.074	1.4		0.147				
		35	0.092	1.4		0.184				
		36	0.072	1.8		0.102				
		37	0.098	1.8		0.139				
		38	0.099	1.8		0.140				
		39	0.125	1.8		0.177				
		40	0.098	2.25		0.105				
		41	0.101	2.25		0.108				
		42	0.139	2.25		0.149				
		43	0.140	2.25		0.150				
		44	0.141	2.25		0.151				
		45	0.160	2.25		0.171				
	**F**	46	0.096	1.8	18	0.137	Perf.	0	0	Material F1 (fine sand and coarse sand, *d*_50_ = 0.98 mm)
		47	0.114	1.8		0.162				
		48	0.114	1.8		0.162				Material F2 (fine sand and coarse sand, *d*_50_ = 0.96 mm)
		49	0.093	1.8		0.132				
		50	0.095	1.8		0.134				Material F3 (fine sand and coarse sand, *d*_50_ = 0.91 mm)
		51	0.115	1.8		0.163				
	**G**	52	0.072	1.8	18	0.102	Perf.	0	-0.10	Material 1 (uniform sand *d*_50_ = 1.03 mm)
		53	0.100	1.8		0.142				
		54	0.120	1.8		0.171				

## References

[1] Baldassarre GD (2015). Debates - Perspectives on Socio-hydrology: Capturing Feedbacks between Physical and Social Processes. Water Resour. Res..

[2] ASCE/EWRI Task Committee on Dam/Levee Breaching (2011). Earthen embankment breaching. J. Hyd. Eng..

[3] Rifai I (2017). Overtopping induced failure of noncohesive, homogeneous fluvial dikes. Water Resour. Res..

[4] Orlandini S, Moretti G, Albertson JD (2015). Evidence of an Emerging Levee Failure Mechanism Causing Disastrous Floods in Italy. Water Resour. Res..

[5] Schiereck, G. Fundamentals on Water Defences. *Rijkswaterstaat*, *DWW* (1998).

[6] Vrijling JK, Schweckendiek T, Kanning W (2011). Safety Standards of Flood Defences. 3rd International Symposium on Geotechnical Safety and Risk..

[7] CIRIA, U.S. Ministry of Ecology, & USACE (United States Army Corps of Engineers). *International levee handbook,* 156–175 (CIRIA, 2013).

[8] FEMA. *Federal guidelines for dam safety: Glossary of terms* (2004).

[9] Vorogushyn S, Merz B, Lindenschmidt K-E, Apel H (2010). A new Methodology for Flood Hazard Assessment Considering Dike Breaches. Water Resour. Res..

[10] Danka, J. & Zhang, L. M. Dike Failure Mechanisms and Breaching Parameters. *J. Geotech. Geoenvir. Eng.* **141** (2015).

[11] Jandora, J. & Říha, J. *The Failure of Embankment Dams Due to Overtopping,* 9–12 (Vutium, 2008).

[12] Rifai Ismail, El Kadi Abderrezzak Kamal, Erpicum Sebastien, Archambeau Pierre, Violeau Damien, Pirotton Michel, Dewals Benjamin (2018). Floodplain Backwater Effect on Overtopping Induced Fluvial Dike Failure. Water Resources Research.

[13] Rifai, I. *et al*. Accompanying dataset for: “Flow and detailed 3D morphodynamic data from laboratory experiments of fluvial dike breaching” *Zenodo*, 10.5281/zenodo.1494800 (2018). 10.1038/s41597-019-0057-y PMC6514018 31086190

[14] Rifai, I. Rupture de digues fluviales par surverse. *PhD Thesis*, (Université Paris-Est et Université de Liège, 2018).

[15] Coleman SE, Andrews DP, Webby MG (2002). Overtopping Breaching of Noncohesive Homogeneous Embankments. J. Hydraul. Eng..

[16] Feliciano Cestero, J. A., Imran, J. & Chaudhry, M. H. Experimental investigation of the effects of soil properties on levee breach by overtopping. *J*. *Hydraul*. *Eng*. **141** (2014).

[17] Frank, P.-J. R. Hydraulics of Spatial Dike Breach. *PhD thesis*, (ETH Zurich, 2016).

[18] Michelazzo G, Oumeraci H, Paris E (2014). New Hypothesis for the Final Equilibrium Stage of a River Levee Breach due to Overflow. Water Resour. Res..

[19] Pickert G, Weitbrecht V, Bieberstein A (2011). Beaching of Overtopped River Embankments Controlled by Apparent Cohesion. J. Hydraul. Res..

[20] Abdel-Aziz, I. Y. & Karara, M. H. Direct linear transformation from comparator coordinates into object space coordinates in close range photogrammetry. *ASP Symposium on Close-Range Photogrammetry*. 1–18 (1971).

[21] Rifai, I. *et al*. Monitoring topography of laboratory fluvial dike models subjected to breaching based on a laser profilometry technique. *River Sedimentation: Proceedings of the 13*^*th*^*International Symposium on River Sedimentation*, 380–386 (2016)

[22] Savitzky A, Golay MJE (1964). Smoothing and Differentiation of Data by Simplified Least Squares Procedures. Anal. Chem..

