# Supplementary material for: Flow and detailed 3D morphodynamic data from laboratory experiments of fluvial dike breaching
Source: Sci Data. 2019 May 13;6:53. doi: 10.1038/s41597-019-0057-y (PMC6514018; doi:10.1038/s41597-019-0057-y)
Supplement: Supplementary file 2 — Supplementary File 1 [file 41597_2019_57_MOESM2_ESM.pdf]

```
HDF5 Test_1.h5

    Group '/Experimental_configuration'

        Dataset 'L_mc'
            Size:  1
            Datatype:   H5T_IEEE_F64LE (double)

        Dataset 'Q_i'
            Size:  1
            Datatype:   H5T_IEEE_F64LE (double)

        Dataset 'Rating_curve_of_Perf._plane_20'
            Size:  133x2
            Datatype:   H5T_IEEE_F64LE (double)

        Dataset 'Z_fp'
            Size:  1
            Datatype:   H5T_IEEE_F64LE (double)

        Dataset 'd_50'
            Size:  1
            Datatype:   H5T_IEEE_F64LE (double)

        Dataset 'l_mc'
            Size:  1
            Datatype:   H5T_IEEE_F64LE (double)

        Dataset 'z_bt'
            Size:  1
            Datatype:   H5T_IEEE_F64LE (double)

    Group '/Hydraulics'

        Dataset 'Q_b'
            Size:  339300
            Datatype:   H5T_IEEE_F64LE (double)

        Dataset 'Q_d'
            Size:  339300
            Datatype:   H5T_IEEE_F64LE (double)

        Dataset 'Q_o'
            Size:  339300
            Datatype:   H5T_IEEE_F64LE (double)

        Dataset 't'
            Size:  339300
            Datatype:   H5T_IEEE_F64LE (double)

        Dataset 'z_G1'
            Size:  339300
            Datatype:   H5T_IEEE_F64LE (double)

        Dataset 'z_G2'
            Size:  339300
            Datatype:   H5T_IEEE_F64LE (double)

        Dataset 'z_G3'
            Size:  339300
            Datatype:   H5T_IEEE_F64LE (double)

        Dataset 'z_G4'
            Size:  339300
            Datatype:   H5T_IEEE_F64LE (double)

        Dataset 'z_G5'
            Size:  339300
            Datatype:   H5T_IEEE_F64LE (double)

    Group '/Reconstruction'

        Dataset 't'
```

```
            Size:  47
            Datatype:   H5T_IEEE_F64LE (double)

        Dataset 'x'
            Size:  305
            Datatype:   H5T_IEEE_F64LE (double)

        Dataset 'y'
            Size:  176
            Datatype:   H5T_IEEE_F64LE (double)

        Dataset 'z'
            Size:  176x305x47
            Datatype:   H5T_IEEE_F64LE (double)

    Group '/Reconstruction_raw'

        Dataset 'Point_cloud_xyz_(t=0s)'
            Size:  97265x3
            Datatype:   H5T_IEEE_F64LE (double)

        Dataset 'Point_cloud_xyz_(t=100s)'
            Size:  21567x3
            Datatype:   H5T_IEEE_F64LE (double)

        Dataset 'Point_cloud_xyz_(t=1020s)'
            Size:  18456x3
            Datatype:   H5T_IEEE_F64LE (double)

        Dataset 'Point_cloud_xyz_(t=110s)'
            Size:  20384x3
            Datatype:   H5T_IEEE_F64LE (double)

        Dataset 'Point_cloud_xyz_(t=120s)'
            Size:  20627x3
            Datatype:   H5T_IEEE_F64LE (double)

        Dataset 'Point_cloud_xyz_(t=12s)'
            Size:  21210x3
            Datatype:   H5T_IEEE_F64LE (double)

        Dataset 'Point_cloud_xyz_(t=1320s)'
            Size:  19131x3
            Datatype:   H5T_IEEE_F64LE (double)

        Dataset 'Point_cloud_xyz_(t=150s)'
            Size:  20438x3
            Datatype:   H5T_IEEE_F64LE (double)

        Dataset 'Point_cloud_xyz_(t=1620s)'
            Size:  18357x3
            Datatype:   H5T_IEEE_F64LE (double)

        Dataset 'Point_cloud_xyz_(t=16s)'
            Size:  20464x3
            Datatype:   H5T_IEEE_F64LE (double)

        Dataset 'Point_cloud_xyz_(t=180s)'
            Size:  21433x3
            Datatype:   H5T_IEEE_F64LE (double)

        Dataset 'Point_cloud_xyz_(t=1920s)'
            Size:  17530x3
            Datatype:   H5T_IEEE_F64LE (double)

        Dataset 'Point_cloud_xyz_(t=20s)'
            Size:  21191x3
            Datatype:   H5T_IEEE_F64LE (double)

        Dataset 'Point_cloud_xyz_(t=210s)'
            Size:  20059x3
            Datatype:   H5T_IEEE_F64LE (double)
```

```
        Dataset 'Point_cloud_xyz_(t=2220s)'
            Size:  18812x3
            Datatype:   H5T_IEEE_F64LE (double)

        Dataset 'Point_cloud_xyz_(t=23s)'
            Size:  21440x3
            Datatype:   H5T_IEEE_F64LE (double)

        Dataset 'Point_cloud_xyz_(t=240s)'
            Size:  20289x3
            Datatype:   H5T_IEEE_F64LE (double)

        Dataset 'Point_cloud_xyz_(t=2520s)'
            Size:  18932x3
            Datatype:   H5T_IEEE_F64LE (double)

        Dataset 'Point_cloud_xyz_(t=270s)'
            Size:  20443x3
            Datatype:   H5T_IEEE_F64LE (double)

        Dataset 'Point_cloud_xyz_(t=27s)'
            Size:  20515x3
            Datatype:   H5T_IEEE_F64LE (double)

        Dataset 'Point_cloud_xyz_(t=2820s)'
            Size:  17442x3
            Datatype:   H5T_IEEE_F64LE (double)

        Dataset 'Point_cloud_xyz_(t=2s)'
            Size:  21176x3
            Datatype:   H5T_IEEE_F64LE (double)

        Dataset 'Point_cloud_xyz_(t=300s)'
            Size:  20848x3
            Datatype:   H5T_IEEE_F64LE (double)

        Dataset 'Point_cloud_xyz_(t=30s)'
            Size:  21409x3
            Datatype:   H5T_IEEE_F64LE (double)

        Dataset 'Point_cloud_xyz_(t=3120s)'
            Size:  17743x3
            Datatype:   H5T_IEEE_F64LE (double)

        Dataset 'Point_cloud_xyz_(t=3350s)'
            Size:  315354x3
            Datatype:   H5T_IEEE_F64LE (double)

        Dataset 'Point_cloud_xyz_(t=34s)'
            Size:  20038x3
            Datatype:   H5T_IEEE_F64LE (double)

        Dataset 'Point_cloud_xyz_(t=360s)'
            Size:  19873x3
            Datatype:   H5T_IEEE_F64LE (double)

        Dataset 'Point_cloud_xyz_(t=37s)'
            Size:  21609x3
            Datatype:   H5T_IEEE_F64LE (double)

        Dataset 'Point_cloud_xyz_(t=41s)'
            Size:  20922x3
            Datatype:   H5T_IEEE_F64LE (double)

        Dataset 'Point_cloud_xyz_(t=420s)'
            Size:  20797x3
            Datatype:   H5T_IEEE_F64LE (double)

        Dataset 'Point_cloud_xyz_(t=45s)'
            Size:  21337x3
            Datatype:   H5T_IEEE_F64LE (double)

        Dataset 'Point_cloud_xyz_(t=480s)'
```

```
                Size:  21163x3
                Datatype:   H5T_IEEE_F64LE (double)

            Dataset 'Point_cloud_xyz_(t=48s)'
                Size:  21307x3
                Datatype:   H5T_IEEE_F64LE (double)

            Dataset 'Point_cloud_xyz_(t=52s)'
                Size:  20757x3
                Datatype:   H5T_IEEE_F64LE (double)

            Dataset 'Point_cloud_xyz_(t=540s)'
                Size:  19391x3
                Datatype:   H5T_IEEE_F64LE (double)

            Dataset 'Point_cloud_xyz_(t=55s)'
                Size:  21324x3
                Datatype:   H5T_IEEE_F64LE (double)

            Dataset 'Point_cloud_xyz_(t=5s)'
                Size:  20467x3
                Datatype:   H5T_IEEE_F64LE (double)

            Dataset 'Point_cloud_xyz_(t=600s)'
                Size:  19835x3
                Datatype:   H5T_IEEE_F64LE (double)

            Dataset 'Point_cloud_xyz_(t=60s)'
                Size:  20727x3
                Datatype:   H5T_IEEE_F64LE (double)

            Dataset 'Point_cloud_xyz_(t=660s)'
                Size:  19716x3
                Datatype:   H5T_IEEE_F64LE (double)

            Dataset 'Point_cloud_xyz_(t=70s)'
                Size:  20173x3
                Datatype:   H5T_IEEE_F64LE (double)

            Dataset 'Point_cloud_xyz_(t=780s)'
                Size:  20274x3
                Datatype:   H5T_IEEE_F64LE (double)

            Dataset 'Point_cloud_xyz_(t=80s)'
                Size:  20277x3
                Datatype:   H5T_IEEE_F64LE (double)

            Dataset 'Point_cloud_xyz_(t=900s)'
                Size:  19527x3
                Datatype:   H5T_IEEE_F64LE (double)

            Dataset 'Point_cloud_xyz_(t=90s)'
                Size:  21591x3
                Datatype:   H5T_IEEE_F64LE (double)

            Dataset 'Point_cloud_xyz_(t=9s)'
                Size:  20913x3
                Datatype:   H5T_IEEE_F64LE (double)

            Dataset 't'
                Size:  47
                Datatype:   H5T_IEEE_F64LE (double)
```
